# Golden*Pi*CS: a Golden Gate-derived modular cloning system for applied synthetic biology in the yeast *Pichia pastoris*

**DOI:** 10.1186/s12918-017-0492-3

**Published:** 2017-12-08

**Authors:** Roland Prielhofer, Juan J. Barrero, Stefanie Steuer, Thomas Gassler, Richard Zahrl, Kristin Baumann, Michael Sauer, Diethard Mattanovich, Brigitte Gasser, Hans Marx

**Affiliations:** 1 Department of Biotechnology, BOKU University of Natural Resources and Life Sciences, Muthgasse 18, 1190 Vienna, Austria; 2 Austrian Centre of Industrial Biotechnology (acib), Vienna, Austria; 3 Present Address: Department of Chemical, Biological, and Environmental Engineering, Escola d’Enginyeria, Universitat Autònoma de Barcelona, Barcelona, Spain; 4 Present Address: Novartis, Vienna, Austria

**Keywords:** *Pichia pastoris*, Cell engineering, Golden Gate cloning, GoldenMOCS, Golden*Pi*CS, Synthetic biology

## Abstract

**Background:**

State-of-the-art strain engineering techniques for the host *Pichia pastoris* (syn. *Komagataella* spp.) include overexpression of homologous and heterologous genes, and deletion of host genes. For metabolic and cell engineering purposes the simultaneous overexpression of more than one gene would often be required. Very recently, Golden Gate based libraries were adapted to optimize single expression cassettes for recombinant proteins in *P. pastoris*. However, an efficient toolbox allowing the overexpression of multiple genes at once was not available for *P. pastoris*.

**Methods:**

With the Golden*Pi*CS system, we provide a flexible modular system for advanced strain engineering in *P. pastoris* based on Golden Gate cloning. For this purpose, we established a wide variety of standardized genetic parts (20 promoters of different strength, 10 transcription terminators, 4 genome integration loci, 4 resistance marker cassettes).

**Results:**

All genetic parts were characterized based on their expression strength measured by eGFP as reporter in up to four production-relevant conditions. The promoters, which are either constitutive or regulatable, cover a broad range of expression strengths in their active conditions (2–192% of the glyceraldehyde-3-phosphate dehydrogenase promoter *P*_*GAP*_), while all transcription terminators and genome integration loci led to equally high expression strength. These modular genetic parts can be readily combined in versatile order, as exemplified for the simultaneous expression of Cas9 and one or more guide-RNA expression units. Importantly, for constructing multigene constructs (vectors with more than two expression units) it is not only essential to balance the expression of the individual genes, but also to avoid repetitive homologous sequences which were otherwise shown to trigger “loop-out” of vector DNA from the *P. pastoris* genome.

**Conclusions:**

Golden*Pi*CS, a modular Golden Gate-derived *P. pastoris* cloning system, is very flexible and efficient and can be used for strain engineering of *P. pastoris* to accomplish pathway expression, protein production or other applications where the integration of various DNA products is required. It allows for the assembly of up to eight expression units on one plasmid with the ability to use different characterized promoters and terminators for each expression unit. Golden*Pi*CS vectors are available at Addgene.

**Electronic supplementary material:**

The online version of this article (10.1186/s12918-017-0492-3) contains supplementary material, which is available to authorized users.

## Background

The yeast *Pichia pastoris* (syn. *Komagataella* spp.) is frequently applied for the production of heterologous proteins, most of which are efficiently secreted [1]. It is also favored for the production of membrane proteins [2], pharmaceuticals and chemical compounds [3] and as a model organism for biomedical research [4]. New genetic tools for *P. pastoris* such as promoters, signal peptides, selection markers, Flp-frt/Cre-lox recombination and CRISPR/Cas9 have been reviewed recently [3]. Compared to other yeast species, *P. pastoris* is distinguished by its methylotrophy, its Crabtree-negative metabolism, its growth to very high cell densities, the low number and concentration of secreted host cell proteins [5] and the availability of many genetic tools and industrially relevant strains (humanized N-glycosylation, protease deficiency) [6]. Genomic integration into specific loci is usually applied by using 5′ and 3′ homologous regions and is crucially depending on the avoidance of repetitive homologous regions and the use of well-purified vector DNA [7].

For recombinant protein production in *P. pastoris*, different highly efficient promoter systems were established (reviewed by Weinhandl et al. [8]) and applied to produce up to several grams per liter of secreted heterologous products, covering proteins intended for biopharmaceutical purposes as well as industrial enzymes [9]. The methanol utilization (MUT) pathway of *P. pastoris* is very efficient and the corresponding genes are highly induced on methanol [10, 11]. MUT promoters (e.g. *P*_*AOX1*_, *P*_*DAS1/2*_ and *P*_*FLD1*_) as well as strong constitutive promoters from highly expressed genes (such as *P*_*GAP*_, derived from the glyceraldehyde-3-phosphate dehydrogenase gene *TDH3* and the promoter of translation elongation factor *P*_*TEF1*_) are frequently applied [12]. Nevertheless, there is still room to further improve productivity and/or protein quality. Besides increasing transcriptional strength by using strong promoters and higher gene copy numbers of the expression cassettes, also cell engineering to boost the host’s folding and secretion capacity or to provide precursors and energy for these processes proved to be beneficial for enhancing product titers (reviewed by Puxbaum et al. [1]). Sometimes such cell engineering approaches require the simultaneous overexpression of more than one gene to reach their full potential (e.g. Nocon et al. [13], Delic et al. [14]). On the other hand, gene knock-outs might be necessary to avoid detrimental processes such as transport to the vacuole or proteolysis (e.g. Idiris et al. [15]). Both of these genetic manipulations require extensive cloning and transformation efforts, which makes them rather time-consuming and tedious.

Today, advanced synthetic biology tools are applied in all fields of microbiology. New cloning methods such as Gateway®, Gibson Assembly and Golden Gate cloning, together with genome editing techniques like CRISPR/Cas9 and TALEN (transcription activator-like effector nucleases), enable efficient and highly specific cell engineering and thereby revolutionized the whole field [16]. Golden Gate cloning is based on type IIs restriction enzymes (which are cutting outside of their recognition sequence) and offers important benefits: it does not require long flanking DNA, it uses efficient one-pot reactions, allows scar-less cloning and is cost-saving compared to many other advanced techniques [17]. In Golden Gate Assembly (GGA), the two different type IIs restriction endonucleases *Bsa*I and *Bpi*I are used which yield four base pair overhangs outside of their recognition sequence. These overhangs can be freely designed and are termed fusion sites (Fs). These fusion sites enable base pair precise assembly of genetic parts such as promoters, coding sequences (CDS) and transcription terminators. By using simultaneous restriction and ligation in efficient one-pot cloning reactions, rapid assembly of multiple DNA fragments is achieved [17].

Recently, Obst et al. [18] and Schreiber et al. [19] reported the use of Golden Gate cloning for the generation of libraries of expression cassettes in *P. pastoris*, which were tested for the production of reporter proteins by the assembly of standardized parts such as promoters, ribosome binding sites, secretion signals and terminators in a fast and efficient way. These studies aimed to optimize a single transcription unit for the production of one heterologous protein of interest (either a fluorescent reporter or an antimicrobial peptide). Vogl et al. [20] used Gibson assembly with a set of MUT-related promoters and novel transcription terminators for the overexpression of multiple genes in *P. pastoris* and could show a strong effect of the inserted promoters when overexpressing the carotenoid pathway (crtE, crtB, crtI, and crtY). Our study extends the versatile Golden Gate technique for all applications in *P. pastoris* where the simultaneous integration of multiple DNA products is required (e.g. cell engineering, pathway expression, protein production, co-expression of cofactors) and aims beyond the mere assembly of single expression cassettes for the heterologous protein of interest.

For this purpose, we adapted the Golden Gate based modular cloning (MoClo) introduced by Weber et al. [21], to create the Golden*Pi*CS (Golden Gate derived *P. pastoris* cloning system) vector toolkit. Golden*Pi*CS is part of a universal system termed GoldenMOCS, standing for Golden Gate-derived Multiple Organism Cloning System [22]. The GoldenMOCS platform enables versatile integration of host specific parts such as promoters, terminators, and resistance cassettes, origins of replication or genome integration loci to adapt the plasmid to the needs of the experiment and the host cell to be engineered. Here, we present the GoldenMOCS- subsystem Golden*Pi*CS designed for use in *P. pastoris* and the characterization of its individual genetic parts using eGFP (enhanced green fluorescence protein) as a reporter. Vectors of these systems were deposited at Addgene as Golden*Pi*CS kit (#1000000133).

## Results and discussion

**Fig. 1:**
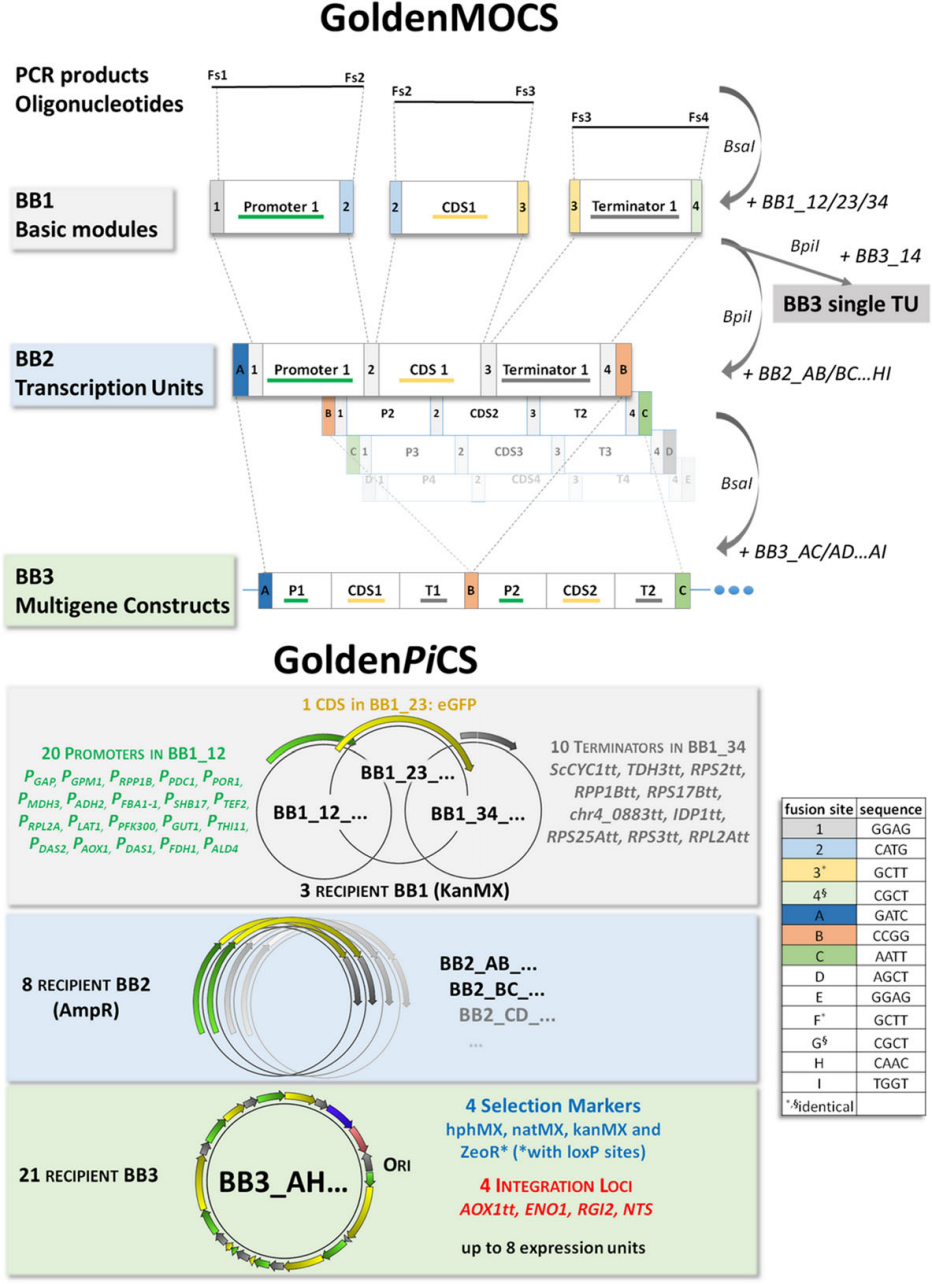
Assembly strategy and hierarchical backbone levels of the cloning systems GoldenMOCS and Golden*Pi*CS. In the microorganism-independent general platform GoldenMOCS, DNA products (synthetic DNA, PCR products or oligonucleotides) are integrated into BB1 by a *Bsa*I Golden Gate Assembly and fusion sites Fs1, Fs2, Fs3 and Fs4. Fusion sites are indicated as colored boxes with corresponding fusion site number or letter. Basic genetic elements contained in backbone 1 (BB1) can be assembled in recipient BB2 by performing a *Bpi*I GGA reaction. The transcription units in BB2 are further used for *Bsa*I assembly into multigene BB3 constructs. Single transcription units can be obtained by direct *Bpi*I assembly into recipient BB3 with fusion sites Fs1-Fs4. Fusion sites determine module and transcription unit positions in assembled constructs. Thereby, fusion sites Fs1 to Fs4 are used to construct single expression cassettes in BB2 and are required between promoter (Fs1-Fs2), CDS (Fs2-Fs3) and terminator (Fs3-Fs4). Fusion sites FsA to FsI are designed to construct BB3 plasmids and separate the different expression cassettes from each other. The FSs are almost randomly chosen sequences and only FS2 has a special function, because it includes the start codon ATG. Golden*Pi*CS additionally includes module-containing BB1s specific for *P. pastoris*: 20 promoters, 1 reporter gene (eGFP) and 10 transcription terminators, and recipient BB3 vectors containing different integration loci for stable genome integration in *P. pastoris* and suitable resistance cassettes (Additional file 2)

Golden*Pi*CS, our Golden Gate-derived *P. pastoris* cloning system, consists of three hierarchical backbone (BB) levels for flexible generation of overexpression plasmids containing multiple transcription units (up to eight per plasmid), four different selection markers and five loci either for targeted genome integration or episomal plasmid maintenance (Fig. 1). We mainly designed the system to enable advanced cell engineering or the expression of whole metabolic pathways. In the lowest cloning level, individual parts such as promoters, coding sequences (e.g. reporters or GOIs) and transcription terminators are incorporated into backbone 1 (BB1) plasmids, which are subsequently assembled to one transcription unit in BB2. This is followed by the assembly of multiple expression units into one BB3, which is designed for subsequent genome integration in *P. pastoris* (four different selection markers and four loci for targeted genome integration are available). Contrary to other cloning techniques, Golden Gate Assembly avoids the need for excessive sequencing, because BB2 and BB3 constructs are assembled by ligation instead of overlap-extension PCR (only BB1 inserts require sequencing after assembly). Correct ligation is assured by defined fusion sites (see Fig. 1): Fusion sites Fs1 to Fs4 are linked to individual parts by PCR and required for their assembly into a transcription unit (BB2) e.g. promoters with coding sequences (‘CATG’, fusion site Fs2). Fusion sites FsA to FsI are used for the assembly of multiple expression units in BB3 e.g. for fusing the first transcription unit to the second (‘CCGG’, fusion site FsB). The different BB3 vectors with fusion sites FsA-FsC, FsA-FsD, to FsA-FsI are designed for the assembly of two, three, … up to eight transcription units in one single plasmid. Internal *Bsa*I and *Bpi*I restriction sites must be removed in all modules by introducing point mutations, with consideration of the codon usage of *P. pastoris*.

**Fig. 2:**
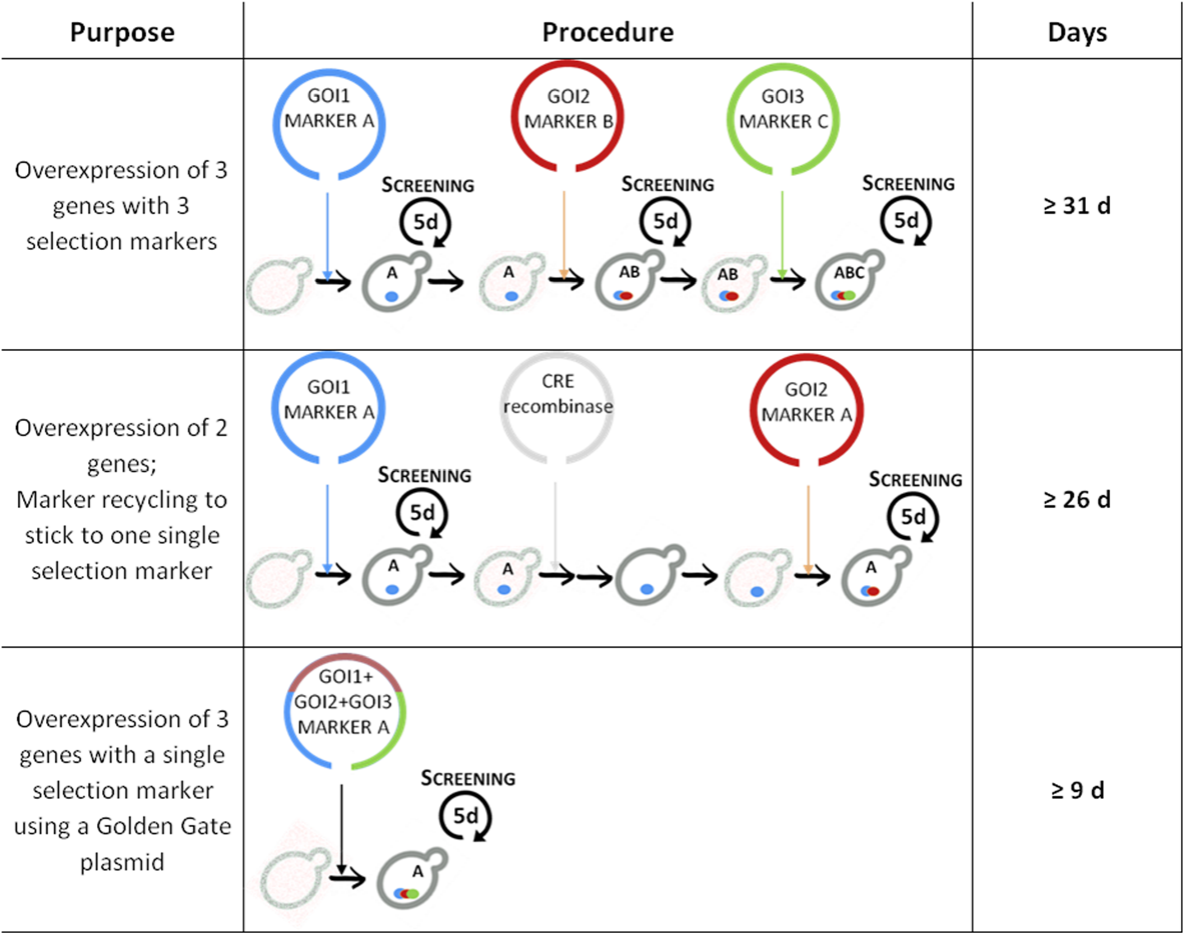
Comparison of conventional and Golden Gate based strain engineering strategies for *P. pastoris*. Overexpression of multiple genes (GOIs) in *P. pastoris* using conventional cloning plasmids requires several rounds of competent making (2 days), transformation (4 days including second streak-out) and clone screening (5 days) and takes at least 31 days for three genes with three selection markers. Alternatively, multiple vectors can be co-transformed at once, but resulting transformation efficiencies are usually very low and clonal variation increases. Appropriate flanking sites for the selection marker (loxP or FRT sites) enable marker recycling by recombinases (Cre or Flp, respectively), but require one more round of competent making and transformation which takes at least eight additional days. Golden Gate plasmids carry several transcription units at once and thereby enable transformation and screening in only nine days

Previously, strain engineering approaches with *P. pastoris* often relied on pGAPz, pPIC6 (Invitrogen) or related expression vectors, which harbor only one transcription unit (Fig. 2). Cloning of concatemers containing more than two transcription units proved to be highly time consuming and also led to unpredictable integration events when using repetitive promoter and terminator sequences [23, 24]. Therefore, conventional overexpression of *n* genes requires *n* cycles of preparing competent cells and transforming them, and the use of *n* different selection markers. For overexpression of three factors plus screenings using consecutive transformations, the procedure would take at least 31 days (four days for transformation and re-streak, five days for screening and two days to prepare competent cells; Fig. 2, upper panel). Furthermore, selection markers can be removed and recycled, with the cost of an additional cycle of competent-making and transformation (Fig. 2, middle panel). In addition to that, the use of different integration loci must be included to prevent ‘loop-out’ incidents of integrated DNA. Alternatively, co-transformation of multiple vectors can be considered, but this requires the use of several independent selection markers, otherwise in our experience transformation efficiency is low and it is very unpredictable if all vectors get integrated into the genome [25]. Our backbone BB3 Golden Gate plasmids can carry multiple transcription units and hence significantly simplify and shorten the procedure to one single transformation step. Integration of multiple transcription units plus screening lasts only nine days (Fig. 2, lower panel). The thereby generated strains benefit from decreased generation numbers and milder selection procedures.

### Recombination events by repetitive sequences disturb full vector integration in *P. pastoris*

Initially, we started with Golden Gate vectors carrying up to four transcription units with the same promoter and transcription terminator (*P*_*GAP*_ and *ScCYC1tt*). High clonal variation prompted us to analyze gene copy numbers (GCN) of integrated genes and we found that individual transcription units were lost. Similar results have been obtained with *P*_*GAP*_ or *P*_*AOX1*_ based multicopy vectors [24], thus showing that incomplete vector integration is not due to the Golden Gate backbone. We positively confirmed post-transformational integration stability for three of the transformants in three consecutive shake flask- batch cultivations without selection pressure (gene copy numbers were stable for more than 15 generations; Additional file 1: Table S1). Therefore, we conclude that repetitive homologous sequences within the expression vector (*P*_*GAP*_ and *ScCYC1tt* sequences) resulted in recombination events (internal ‘loop out’) during transformation.

The high occurrence of incomplete vector integration prompted us to establish a collection of 20 different promoters and 10 transcription terminators (Table 1), in order to avoid repetitive sequences when creating constructs carrying multiple expression units. Promoters and terminators were selected based on the transcriptional regulation and expression strength of their natively controlled genes in published microarray experiments [26]. All of them were screened in several production-relevant conditions (Additional file 1: Table S2). In addition to established promoters [3, 12], we selected novel yet uncharacterized promoter sequences based on their expression behavior in transcriptomics data from *P. pastoris* cells cultivated on different carbon sources [26]. By applying this collection we aimed to gain the ability to fine-tune the expression of integrated genes. Ideally, promoters for cell engineering purposes should cover a wide range of expression strengths and allow constitutive as well as tunable expression. Transcription terminators were selected from constitutively highly expressed genes [26], many of them being derived from ribosomal protein genes. Also ribosomal genes were reported to be regulated at the level of mRNA stability [27], which is one of the main functions of the 3’UTR contained in the transcription terminator fragments.

**Table 1:** Promoters and terminators of the *P. pastoris* Golden*Pi*CS toolbox

Promoter/Terminator	Short Name	Gene name	ORF	length [bp]	μA mean exp. G	μA mean exp. X	μA mean exp. D	μA mean exp. M	rel. eGFP [%] G	rel. eGFP [%] X	rel. eGFP [%] D	rel. eGFP [%] M
*P*_*GAP*_	*TDH3*	glyceraldehyde-3-phosphate dehydrogenase	PP7435_Chr2–0858	496	175,734	153,293	190,970	89,048	100	100	100	100
*P*_*GPM1*_	*GPM1*	phosphoglycerate mutase	PP7435_Chr3–0360	404	118,686	98,941	165,884	52,844	25	13	26	11
*P*_*RPP1B*_	*RPP1B*	ribosomal protein P1 beta	PP7435_Chr4–0276	999	168,241	88,081	147,457	134,220	22	14	23	13
*P*_*PDC1*_	*PDC1*	pyruvate decarboxylase 1	PP7435_Chr3–1042	989	95,047	34,921	188,242	24,093	19	17	23	16
*P*_*POR1*_	*POR1*	mitochondrial porin	PP7435_Chr2–0411	704	94,576	173,321	58,699	118,862	46	28	19	51
*P*_*MDH3*_	*MDH3*	peroxisomal malate dehydrogenase	PP7435_Chr4–0136	979	26,497	206,905	12,554	87,661	76	83	17	110
*P*_*ADH2*_	*ADH2*	alcohol dehydrogenase 2	PP7435_Chr2–0821	820	117,961	165,203	140,678	61,540	33	47	16	54
*P*_*FBA1–1*_	*FBA1–1*	fructose 1,6-bisphosphate aldolase	PP7435_Chr1–0374	970	152,990	127,354	213,816	57,961	20	11	14	12
*P*_*SHB17*_	*SHB17*	sedoheptulose bisphosphatase	PP7435_Chr2–0185	995	24,473	43,866	35,852	167,426	0	0	0	53
*P*_*TEF2*_	*TEF2*	translational elongation factor EF-1 alpha	PP7435_Chr1–1535	572	184,546	96,226	180,287	161,445			53	
*P*_*RPL2A*_	*RPL2A*	ribosomal 60S subunit protein L2A	PP7435_Chr4–0909	949	184,025	96,715	164,082	143,041			10	
*P*_*LAT1*_	*LAT1*	dihydrolipoamide acetyltransferase	PP7435_Chr1–0349	1006	24,576	22,867	31,661	16,960			6	
*P*_*PFK300*_	*PFK300*	Gamma subunit of 6-phosphofructokinase	PP7435_Chr4–0686	229	20,845	18,516	21,844	15,474			2	
*P*_*GUT1*_	*GUT1*	glycerol kinase	PP7435_Chr4–0173	1305	31,172	88,114	1324	12,564	44		1	
*P*_*THI11*_	*THI11*	involved in synthesis of thiamine precursor	PP7435_Chr4–0952	1005	77,578	101,948	45,756	120,542			36	
*P*_*DAS2*_	*DAS2*	dihydroxyacetone synthase 2	PP7435_Chr3–0350	1005	1316	32,401	1268	186,477			0	160
*P*_*AOX1*_	*AOX1*	alcohol oxidase 1	PP7435_Chr4–0130	928	5301	159,461	4487	188,502			0	105
*P*_*DAS1*_	*DAS1*	dihydroxyacetone synthase 1	PP7435_Chr3–0352	554	2518	45,547	2200	239,261			0	99
*P*_*FDH1*_	*FDH1*	formate dehydrogenase	PP7435_Chr3–0238	1032	3126	215,618	2418	179,036			0	83
*P*_*ALD4*_	*ALD4*	mitochondrial aldehyde dehydrogenase	PP7435_Chr2–0787	869	21,239	109,237	9330	52,434			0	18
*ScCYC1tt*	YJR048W	cytochrome C isoform 1	Sc: YJR048W	285	–	–				100	100	100
*TDH3tt*	*TDH3*	glyceraldehyde-3-phosphate dehydrogenase	PP7435_Chr2–0858	210	175,734	153,293	190,970	89,048		139	128	126
*RPS2tt*	*RPS2*	protein component of the 40S ribosome	PP7435_Chr1–1396	476	161,620	77,215	151,802	135,407		136	129	128
*RPP1Btt*	*RPP1B*	ribosomal protein P1 beta	PP7435_Chr4–0276	507	168,241	88,081	147,457	134,220		120	117	113
*RPS17Btt*	*RPS17B*	ribosomal protein 51	PP7435_Chr2–0491	495	122,828	89,591	125,205	117,162		124	122	118
*chr4_0883tt*	chr4_0883	not conserved hypothetical protein	PP7435_Chr4–0069	483	127,795	93,922	95,185	113,403		88	99	84
*IDP1tt*	*IDP1*	mitochondrial NADP-specific isocitrate dehydrogenase	PP7435_Chr1–0546	503	11,349	8309	19,236	10,932		126	123	118
*RPS25Att*	*RPS25A*	protein component of the 40S ribosome	PP7435_Chr2–0346	493	146,211	100,973	140,035	126,110		140	126	123
*RPS3tt*	*RPS3*	protein component of the 40S ribosome	PP7435_Chr1–0118	174	143,928	59,278	143,257	121,372		137	125	123
*RPL2Att*	*RPL2A*	ribosomal 60S subunit protein L2A	PP7435_Chr4–0909	504	184,025	96,715	164,082	143,041		102	102	93

Mean expression from microarray (μA) data were extracted from [26]. The relative eGFP expression is related to expression under control of P_GAP_ in the respective condition

*G* 2% glycerol, *X* glucose feed bead (limited glucose), *D* 2% glucose, *M* methanol

### Validation of Kozak sequence mutations in the P_*GAP*_ sequence of *P. pastoris*

**Fig. 3:**
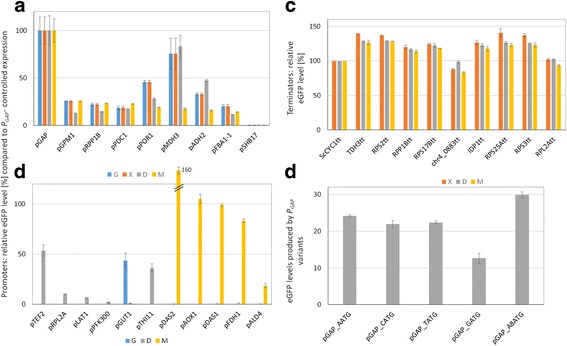
Relative eGFP levels obtained with various elements of the Golden*Pi*CS toolbox. Expression strength of different promoters in comparison to *P*_*GAP*_ tested on different carbon sources (**a**, **b**), expression levels for *P*_*GAP*_-controlled expression in combination with different transcriptional terminators (**c**), and comparison of *P*_*GAP*_ variants with alternative ‘-1’ nucleotides (**d**). At least 10 *P. pastoris* clones were screened to test promoter and terminator function in up to four different conditions: glycerol and glucose excess as present in batch cultivation (“G”, “D”), limiting glucose (“X”) and methanol feed (“M”), both representing fed batch. *P*_*GAP*_ to *P*_*SHB17*_ were tested in ‘G’, ‘D’, ‘X’ and ‘M’ (A). *P*_*TEF2*_ to *P*_*PFK300*_ were validated in ‘D’, while *P*_*GUT1*_, *P*_*THI11*_ and MUT-related promoters were tested in putative repressed and induced conditions (‘D’/‘G’, ‘D’+/−100 μM thiamine and ‘D’/M, respectively) (B). *P*_*GAP*_ variants with alternative ‘-1’ bases were analyzed in glucose excess (‘D’). Relative eGFP levels are related to *P*_*GAP*_- controlled expression (A, B), terminator *ScCYC1tt* (B), or presented as relative value (C)

**Fig. 4:**
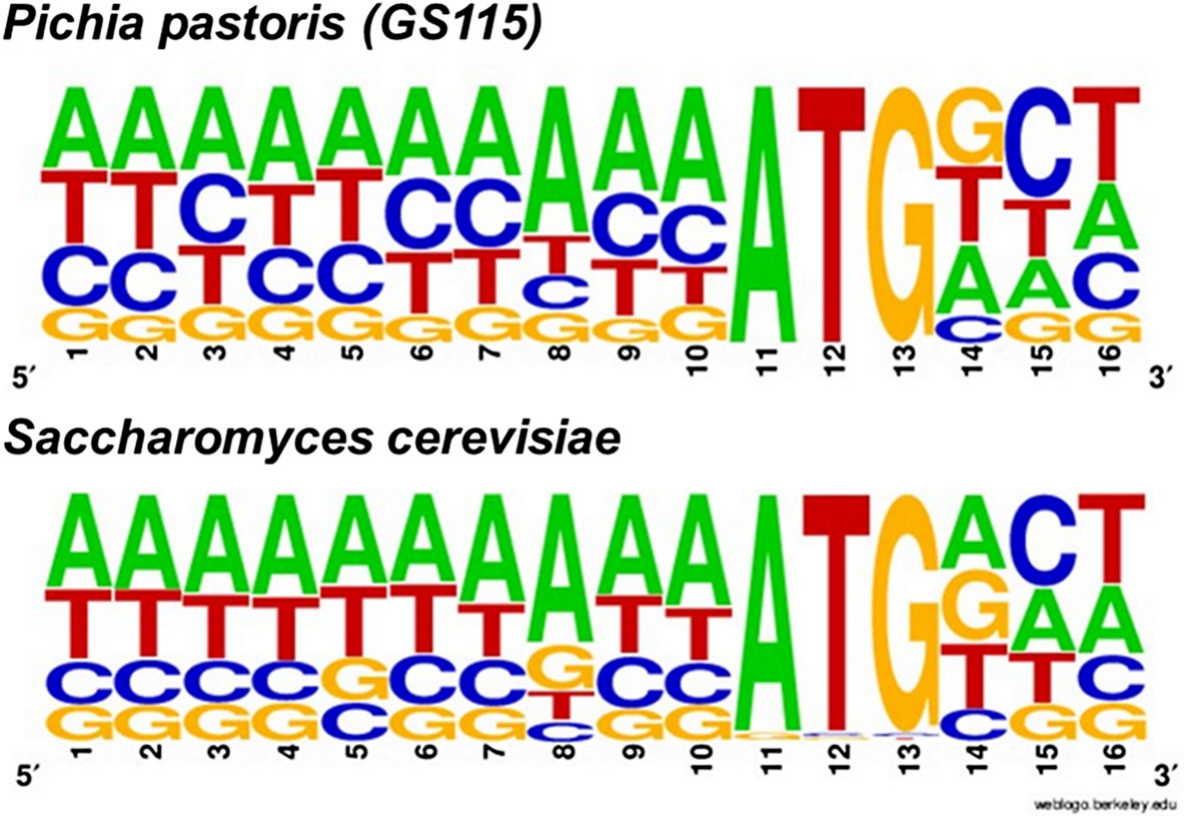
Kozak consensus sequences of *P. pastoris* and *S. cerevisiae*. The sequences were retrieved by the regulatory sequence analysis tool (RSAT) and illustrated using weblogo

In the GoldenMOCS setup, fusion site Fs2 that links the promoter to the GOI contains the start codon ATG and part of the Kozak sequence, which is important for translational initiation in eukaryotes. As this fusion site is a fixed variable in the Golden Gate system, we evaluated the effect of the ‘-1’ position of the *P*_*GAP*_ promoter (position in front of the start codon, Fig. 3d) on reporter gene expression in *P. pastoris*. Due to the ‘CATG’ fusion site, the native ‘A’ in position ‘-1’ of the *P*_*GAP*_ promoter (‘-8’ to ‘-1’: AAAACAC**A**) is changed to ‘C’ (AAAACAC**C**). While *P*_*GAP*_ variants with ‘A’, ‘T’ and ‘C’ at position ‘-1’ performed similarly, the variant with ‘G’ resulted in a lower eGFP level (*P*_*GAP*__GATG; about 40% lower compared to the other variants). The Kozak consensus sequence of *P. pastoris* was analyzed and found to be similar to that of *S. cerevisiae*, which is rich in ‘A’ and poor in ‘G’ bases (Fig. 4). Based on these results, eight bases of the A-rich Kozak consensus sequence were tested in an additional *P*_*GAP*_ variant (*P*_*GAP*__A_8_ATG; position ‘-4’ and ‘-2’ replaced by ‘A’: AAAAAAA**A**) and a slightly increased expression of eGFP was found (Fig. 3d). Nevertheless, we chose the ‘CATG’ fusion site for our Golden*Pi*CS system and kept the ‘GCTT’ fusion site for the GOI-terminator assembly.

### Analysis of Golden*Pi*CS promoter and terminator strength and regulation using eGFP

All promoters and terminators were characterized for their capacity for expression of the intracellular reporter eGFP in appropriate conditions (Additional file 1: Table S2): glycerol or glucose excess (“G” and “D”, respectively, maximum specific growth rate μ_MAX_~0.22 h^−1^) as present in batch cultivation, limiting glucose (“X”, 12 mm glucose feed beads, specific growth rate μ~ 0.04 h^−1^) and methanol feed (“M”, μ_MAX_ up to 0.1 h^−1^), the latter two representing conditions as encountered during fed batch cultivation. *P*_*GAP*_ was used as reference to evaluate the expression strength of the promoters. *P*_*TEF2*_, *P*_*GPM1*_, *P*_*RPP1B*_, *P*_*PDC1*_, *P*_*POR1*_, *P*_*ADH2*_, *P*_*FBA1*–1_, *P*_*RPL2A*_, *P*_*LAT1*_, *P*_*PFK300*_ and *P*_*MDH3*_ were confirmed to have a constitutive regulation with a range of eGFP expression of 2–192% of *P*_*GAP*_ in all tested conditions (Fig. 3a and b). Promoters responsive to thiamine (*P*_*THI11*_), glycerol (*P*_*GUT1*_) and methanol (*P*_*AOX1*_, *P*_*DAS1*_, *P*_*DAS2*_, *P*_*FDH1*_, *P*_*SHB17*_ and *P*_*ALD4*_) were well repressed (0% of *P*_*GAP*_) and induced (to 18–160% of *P*_*GAP*_) in the repressed and induced conditions, respectively (Table 1 and Fig. 3b). The selected transcription terminator sequences did not have large effects on eGFP expression levels (tested with *P*_*GAP*_, normalized to termination with *ScCYC1tt*, Table 1 and Fig. 3c). However, eGFP levels were about 20% lower compared to the other terminators when using *ScCYC1tt* (also reported recently in [7]), *chr4_0883tt* and *RPL2Att*. Recently, a set of transcriptional terminators derived from MUT- and other metabolic genes of *P. pastoris* was tested in combination with expression under control of P_*AOX1*_ and a broader range of expression levels from 60 to 100% relative to the *AOX1* terminator was observed [20]. Compared to that, transcription terminators of Golden*Pi*CS, which were mainly selected from ribosomal genes (reported to be regulated at the level of mRNA stability [27]), appear to result in more uniform expression levels.

### Evaluation of genome integration efficiency of Golden*Pi*CS multigene constructs

**Fig. 5:**
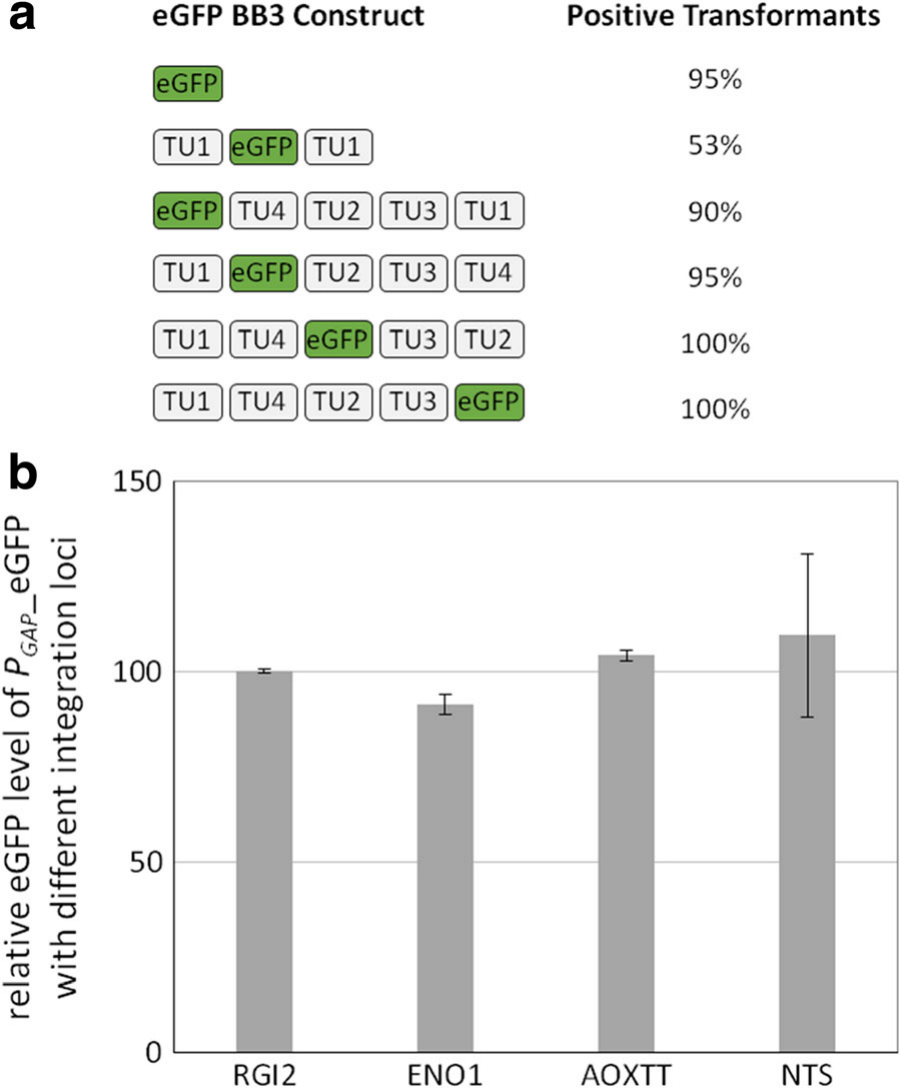
Genome integration efficiency of Golden Gate vectors without repetitive homologous sequences (**a**) and expression of eGFP obtained after integration into different genomic loci **(b).** Genome integration efficiency (fraction of positive clones) are shown for *P. pastoris* transformed with different Golden Gate vectors containing *P*_*GAP*_-eGFP_*ScCYC1tt*: control plasmid (single eGFP), eGFP in position 2 with a repetitive transcription unit (TU1) in position 1 and 3 (‘loop-out’ control) and 4 quadruple combinations with eGFP in position 1, 2, 3 and 5 next to four other transcription units TU1–4) (a). Genome integration was verified by analyzing eGFP expression of each 16 (controls) and 22 clones (quadruple combinations), respectively. TU1–4 were cloned with the promoters and transcription terminators P_*POR1*_/*RPS3tt*, P_*PDC1*_/*IDP1tt*, P_*ADH2*_/*RPL2Att* and P_*MDH3*_/*TDH3tt*, respectively. Coding sequences of TU1–4 were derived from various non-essential intracellular-protein coding genes of *P. pastoris* with a length of 500–2000 bp. The influence of the genomic integration locus was analyzed using vectors containing only one transcription unit (*P*_*GAP*__eGFP_*ScCYC1tt*) (b). In both cases clones were screened under glucose surplus

Vectors containing up to five transcription units without repetitive homologous sequences, including *P*_*GAP*__eGFP_*ScCYC1tt* in different positions of the vector as readout, resulted in complete vector integration for more than 97% of all *P. pastoris* transformants, although we observed a slight efficiency decrease with increasing distance from the selection marker (Fig. 5a). Relative eGFP levels were very similar for all tested constructs. In contrast, just 56% eGFP positive clones were obtained when a control vector containing twice the identical transcription unit with *P*_*GAP*__eGFP_*ScCYC1tt* in between was used (only 9 out of 16 clones contained an integrated copy). To further increase our repertoire for strain engineering purposes, Golden Gate constructs containing a single eGFP transcription unit (*P*_*GAP*__eGFP_*ScCYC1tt*) targeted to different integration loci (*AOX1tt*, *RGI2*, *ENO1* or *NTS*) were analyzed. The eGFP levels were similar with all tested constructs expressing from different genomic loci in *P. pastoris* (Fig. 5b).

### Example of multi-gene construct assembly with Golden*Pi*CS

**Fig. 6:**
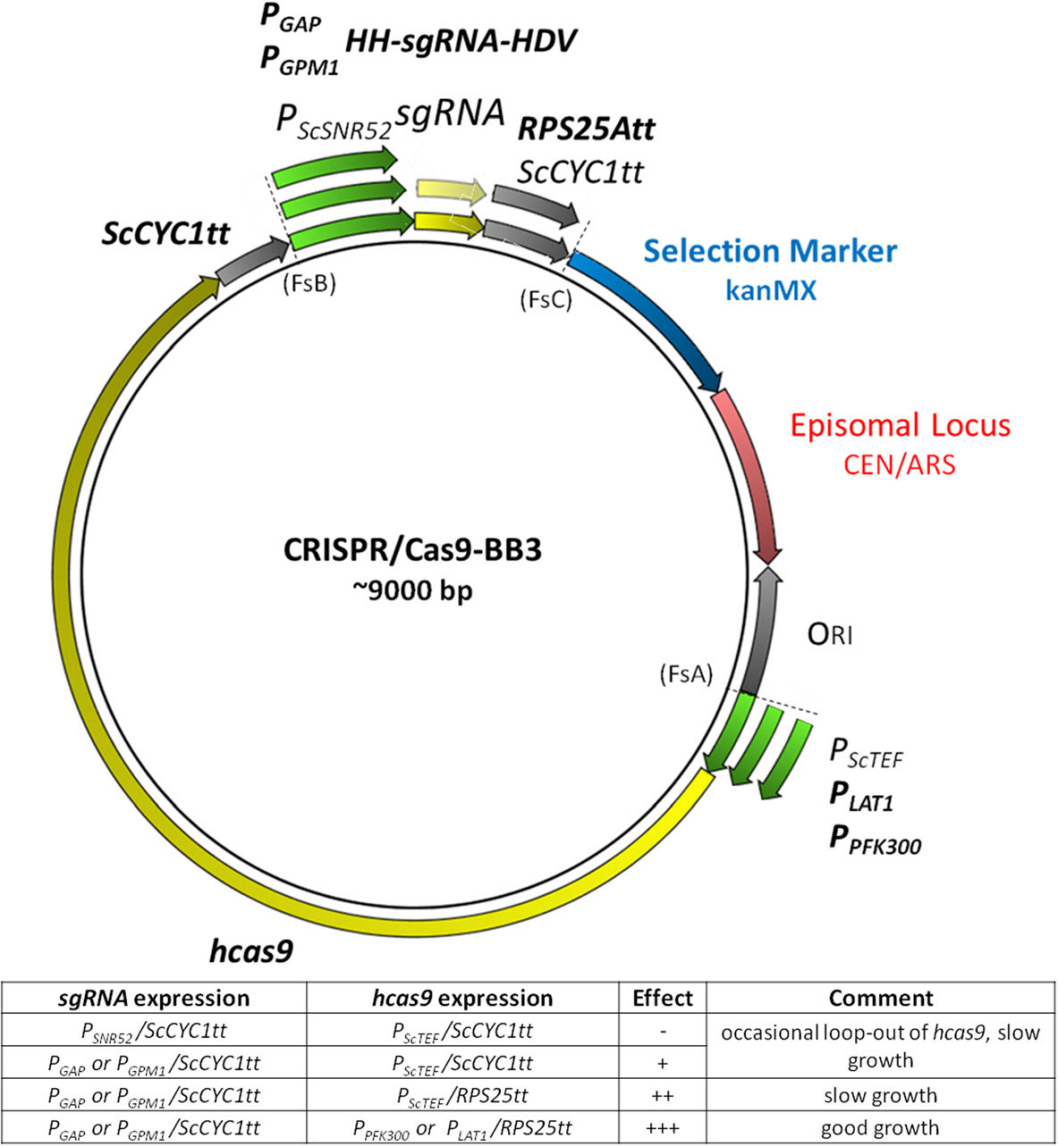
Overview of CRISPR/Cas9-BB3 plasmids assembled using Golden*Pi*CS. Transcription units for *sgRNA* (fusion of crRNA and tracrRNA [42]) and *hcas9* (with SV40 nuclear localization sequence at its C-terminus) with various promoters and transcription terminators were assembled in BB3cK_AC (CEN/ARS locus, KanMX selection marker, fusion sites FsA-FsC). The plasmids were successfully used to generate InDel mutations in different genomic loci and eGFP in *P. pastoris*. The effects of the tested constructs are summarized in the table below the vector scheme. Modules which worked most efficiently are indicated in bold in the vector scheme

Efficient genome editing by the CRISPR/Cas9 system was shown in many organisms including *P. pastoris* [28]. However, efficiencies and applicability were not uniformly high when using different targets or approaches. We applied Golden*Pi*CS to assemble different alternatives of the two transcription units of humanized Cas9 (*hcas9*) and single guide RNA (*sgRNA*) on one single episomal plasmid and test them for their efficiency to perform InDel mutations in *P. pastoris* (Fig. 6). The assembled BB3 plasmids were episomally maintained in *P. pastoris* by using the *S. cerevisiae* CEN/ARS locus instead of a genome integration locus [29]. Initially, we tested *sgRNA* expression with the *SNR52* promoter (RNAPIII promoter capable to express non-coding RNA) and the *SUP4* terminator from *S. cerevisiae* [30], and *hcas9* controlled by *P*_*ScTEF*_ and *ScCYC1tt*, but we could not obtain InDel mutations in *P. pastoris*. Next, we tried the strong RNAPII promoter *P*_*GAP*_ and flanking self-splicing hammerhead (HH, 5′) and hepatitis delta virus (HDV, 3′) ribozyme sequences for correct processing of the *sgRNA* [31] and observed an efficiency of up to 90% when targeting eGFP, similar as described by Weninger et al. [28]. To reduce potential loop-out problems of the Cas9 transcription unit encountered during expression in *P. pastoris*, we exchanged the *ScCYC1tt* terminator of the *sgRNA* transcription unit for the *P. pastoris*-derived transcription terminator *RPS25Att* to avoid repetitive sequences. Regarding the expression of *hcas*9, we obtained similarly high efficiencies with different promoters, however, growth was weaker with *P*_*ScTEF1*_ while it was almost unaffected when using *P*_*LAT1*_ or *P*_*PFK300*_. Targeting efficiency was mostly dependent on the applied *sgRNA* sequence, as we found large differences for several examples: At least two different *sgRNA*s designed by CHOP CHOP [32] were tested for each target. In all cases, they resulted in different efficiencies for InDel formation, e.g. two different *sgRNA*s each targeting *AOX1* and *DAS2*, which are non-essential on glucose, resulted in largely different efficiencies of 38% vs. 100% and 0% vs. 100%, respectively. Therefore, we recommend to test at least two different *sgRNA*s for each target sequence.

With this example we demonstrate the suitability of Golden*Pi*CS to assemble several expression cassettes on one vector and to rapidly create new variants by exchanging parts like promoters, terminators or expression sequences. The Golden*Pi*CS based vectors for the described CRISP/Cas9 approach are available as separate kit at Addgene (Gassler et al. 2018.) This allowed rapid optimization of a CRISPR/Cas9-BB3 for efficient InDel mutations in *P. pastoris* within a short time.

## Conclusions

Advanced synthetic biology tools revolutionized genetic engineering and are applied for many different organisms today. Strain engineering of *P. pastoris* has been shown to improve bottlenecks of protein synthesis, folding and secretion [1, 6]. Recently, we reported that overexpression of different pentose phosphate pathway genes had synergistic effects on production of human superoxide dismutase in *P. pastoris* [13], as were combinations of individual enzymes involved in redox homeostasis and oxidative protein folding [14]. For such complex cell engineering approaches more efficient strategies are needed. Recently, the carotenoid pathway was introduced into *P. pastoris* and fine-tuned by using a set of MUT-related promoters and terminators constructed by Gibson assembly [20, 33]. At about the same time, we established Golden*Pi*CS, a Golden Gate based modular cloning system for genetic engineering of *P. pastoris*. Both systems facilitate the assembly of multiple transcription units with the possibility to fine-tune the expression of each target individually. In our opinion, Golden Gate cloning has crucial advantages such as its low price, broad flexibility and high efficiency. These advantages have inspired several research groups to apply Golden Gate cloning for their purposes. So far, Golden Gate based screening in *P. pastoris* was dedicated to design and optimize the expression of a single recombinant gene for its production in *P. pastoris* by high throughput testing of different promoters and secretion signals [18, 19]. In contrast, our Golden*Pi*CS system is aimed to facilitate cell engineering by allowing the overexpression of multiple genes e.g. redox partners or metabolic enzymes that act in a common pathway. Therefore, our system allows for the assembly of up to eight expression units on one plasmid with the ability to use different characterized promoters and terminators for each expression unit. The latter was proven to be essential to obtain stable transformants. The toolbox described by Obst et al. [18] is based on the yeast toolkit (YTK) and is primarily designed to test gene expression of a heterologous protein of interest with different regulatory elements (with a strong focus on the comparison of different published signal peptides), but is rather limited to a subset of 4–6 strong promoters and just employs two transcription terminators, one thereof taken from the original YTK. It also overlaps with the high throughput screening platform described by Schreiber et al. [19], where two promoters and three different secretion signals were tested for the production of antimicrobial plasmids. The Golden*Pi*CS system is not primarily aimed for the expression screening of the heterologous protein itself (although it can be used for it), but dedicated to combinatorial cell engineering strategies or the expression of whole metabolic pathways. We have thus added an example on the assembly of CRISPR/Cas9 vectors with 2 expression cassettes, where the issue of stability with repeated sequences (and its solution with the promoter library) is illustrated, as well as the advantage of fast assembly of elements. Aside from the development of the Golden*Pi*CS toolkit, we have invested effort to assay promoter and terminator strength in different conditions, to validate the effect of the ‘-1’ position in front of the start codon and to present data for the ‘loop-out’ effect in *P. pastoris* transformants. Importantly, we found that repetitive sequences on the expression vector lead to unwanted recombination events and therefore must be avoided.

Overall, we present the hierarchical multi-organism modular cloning system (GoldenMOCS) and provide several modules and plasmids for *P. pastoris:* Golden*Pi*CS consists of 20 *P. pastoris* promoters, 10 terminators (all *P. pastoris*-derived, except for the terminator *ScCYC1tt*), 4 integration loci (*RGI2*, *ENO1*, *NTS* and *AOX1tt*) and one locus for episomal plasmid maintenance, as well as 4 resistance marker cassettes (hphMX, natMX, kanMX and ZeoR; the latter with loxP sites). With the currently available set of fusion sites, assembly of up to eight expression units per plasmid is possible. All of these are available through Addgene (please note that the ARS/CEN locus for episomal plasmid maintenance is only part of the CRISPR/Cas9 kit; Gassler et al. 2018) and allow high throughput assembly of multigene constructs for cell and metabolic engineering purposes.

## Methods

### Strains and growth conditions

*Escherichia coli* DH10B (Invitrogen) was used for plasmid amplification. Promoter and terminator studies were done in *P. pastoris* (*Komagataella phaffii*) CBS7435(Mut^S^), obtained from Helmut Schwab, Graz University of Technology, Austria. *P. pastoris* clones were screened in 24- deep well plates (Whatman, UK) using appropriate media (complex YP media or synthetic M2 screening media) [4] and selection markers (Additional file 1: Table S3). Plasmids were linearized within the genome integration locus (not applied for the episomal CRISPR/Cas9-BB3’s) and transformed into electro-competent *P. pastoris* by electroporation (2 kV, 4 ms, GenePulser, BioRad) according to [4].

### Molecular biology

Primer design and in silico cloning was performed using the CLC Main Workbench Version 7.7.3. Golden*Pi*CS module sequences and backbones are listed in Additional file 2. Custom DNA oligonucleotides and gBlocks (from IDT, BE), restriction enzymes, T4 Ligase, Q5 polymerase (all from New England Biolabs, DE, or Fermentas, DE) and DNA cleanup kits (from Qiagen, DE, and Promega, DE) were used for routine cloning work.

## GoldenMOCS and Golden*Pi*CS

### Basic principle and background

Golden Gate cloning [17, 34, 35], a modular cloning system, was set up for simultaneous overexpression of multiple genes independent of the microorganism and further developed for application in *P. pastoris* (see Fig. 1 for a schematic overview). We termed the basic system GoldenMOCS, (Golden Gate-derived Multiple Organism Cloning System) and the subsystem specialized for application in *P. pastoris* was named Golden*Pi*CS (includes GoldenMOCS plus further developments). The system is generally comprised of three backbone (BB) levels. BB1 constructs harbor the three basic modules (promoters, coding sequences and terminators), BB2 constructs are used to assemble transcription units (promoter + CDS + terminator) and BB3 are used to further combine multiple transcription units (Fig. 1).

Golden Gate cloning employs type IIs restriction enzymes (*Bsa*I and *Bpi*I) which cut outside of their recognition site and enables scarless cloning, assembly of multiple DNA fragments and efficient one-pot cloning reactions (simultaneous restriction and ligation; termed Golden Gate assembly reaction). Therefore, internal *Bsa*I and *Bpi*I restriction sites must be removed from all modules by introducing point mutations, respecting the codon usage of the host organism (*P. pastoris* codon usage reported by De Schutter et al. [36]).

### Fusion sites, modules and plasmids (summarized in Fig. 1 and Additional file 2)

Golden Gate cloning applies the two type IIs restriction endonucleases *Bsa*I and *Bpi*I, which yield four base pair overhangs outside of their recognition sequence. These overhangs - termed fusion sites (Fs) - can be freely designed and are used to systematically assemble modules in the GoldenMOCS.

Modules of the Golden*Pi*CS include basic modules (promoters, CDSs, terminators), resistance cassettes, integration sites, and linkers (containing restriction sites for DNA integration and excision; BB2 linkers additionally contain a 5′ located strong artificial transcriptional terminator (BBa_B1007, modified *E. coli* thr terminator) to prevent transcriptional read-through from the resistance gene). The following nomenclature is used for basic modules: *P*_*XXXn*_ or pXXXn for promoters, *YYYn* for coding sequences and *ZZZntt* for terminators. Modules from other organisms are indicated by the initials of the species name, e.g. ‘*Sc*’ for *S. cerevisiae*.

Recipient BB1 and BB2 plasmids were adapted from pIDT-SMART (IDT, BE) and pSTBlue-1 (VWR, DE), respectively (with kanamycin/ampicillin resistance). All recipient backbones are comprised of an origin of replication for *E. coli*, a resistance cassette (for *E. coli* or *P. pastoris*, a linker with *Bpi*I and/or *Bsa*I cloning sites, while BB3 additionally contains a genomic locus for integration or episomal plasmid maintenance for *P. pastoris*.

### Golden Gate assembly – BB1

The three basic modules promoter, CDS and terminator are assembled into BB1 using primers with *Bsa*I sites and two appropriate fusion sites: Fs1-Fs2 to integrate into recipient BB1_12 (for promotor modules), Fs2-Fs3 to integrate into BB1_23 (for CDS modules) and Fs3-Fs4 to integrate into BB1_34 (for terminator modules). Multiple fragments (e.g. to introduce mutations or to create fusion genes) can be assembled in the BB1 assembly reaction by appropriate fusion site design. All inserts which were assembled into BB1 need to be checked by sequencing.

### Golden Gate assembly – BB2

Single transcription units (promoter, CDS, terminator) are assembled into a recipient BB2 using the fusion sites Fs1, Fs2, Fs3 and Fs4. Depending on the intended position of the transcription unit in BB3, the appropriate BB2 is used: BB2_AB with FsA-FsB for the first, BB2_BC with FsB-FsC for the second position, etc.

### Golden Gate assembly – BB3

Multiple transcription units are assembled into a recipient BB3 using fusion sites appropriate for the number of transcription units (e.g. A-C for two transcription units). For overexpression of a single transcription unit, direct cloning from BB1 into a special BB3, equipped with a BB2 linker with *Bpi*I restriction sites and fusion sites Fs1-Fs4 (e.g. BB3aN_14) can be done.

### BB3 creation

De novo assembly of BB3 plasmids can be done using a *Bpi*I Golden Gate Assembly reaction with the following modules: linker, resistance cassette, integration locus and Ori modules - including appropriate flanking fusion sites (Fs1–2, Fs2–3, Fs3–4 and Fs4–1, respectively). In order to create recipient BB3 for direct cloning with BB1, the linker with fusion sites FsA-FsB can be replaced using a *Bsa*I reaction with BB2_AB – thereby introducing the BB2 linker with *Bpi*I restriction sites and fusion sites Fs1-Fs4. Amplification and sequencing primers are included in Additional file 2.

BB3 plasmids for CRISPR/Cas9-mediated genome editing were assembled as usual recipient BB3, consisting of resistance cassette, Ori, CEN/ARS locus and a linker containing fusion sites FsA-FsC for integration of two transcription units (Additional file 2 and Fig. 6).

### Golden Gate assembly reaction

One μL *Bsa*I or *Bpi*I (10 U), 40 U T4 Ligase (0.1 μL), 2 μL CutSmart™ Buffer (10×, NEB), 2 μL ATP (10 mM, NEB) and 40 nM dilutions of PCR fragments and/or carrier and recipient backbone were diluted in 20 μL total volume and incubated as follows: 8 to 50 cycles (depending on insert number) of each 2 min at 37 °C and 16 °C, followed by 10 min at 37 °C, 30 min at 55 °C and 10 min at 80 °C (final ligation, digestion and heat inactivation).

#### Characterization of genetic parts in *P. pastoris*

For evaluation of promoter and terminator function (screening), *P. pastoris* transformants were cultivated at 25 °C on a rotary shaker at 280 rpm. Screening conditions were designed to represent bioreactor cultivation phases (Additional file 1: Table S2). Briefly, glycerol and glucose excess conditions (“G”, “D”) as present in batch cultivation were analyzed at a high growth rate of μ_MAX_~0.22 and an OD_600_ of about 3–8. Limiting glucose (“X”, 12 mm glucose feed beads, releasing glucose at a non-linear rate of 1.63 ∙ t^0.74^ mg per disc, Kuhner, CH) and methanol feed (“M”), representing fed batch conditions, were measured at an OD_600_ of about 10 and growth rates around 0.04 h^−1^ and μ_MAX-MeOH_ (up to 0.1 h^−1^), respectively. Growth rates and biomass increase can roughly be calculated from the substrate yield coefficient, which is Y_X/S_ ~ 0.5 for μ > 0.05 h^−1^ on glucose [37] and Y_X/S_ ~ 0.6 on glycerol [38], while it is lower on sole methanol (Y_X/S_ ~ 0.4) and methanol culture lag phases are prolonged [39].

#### Flow cytometry

Analysis of eGFP levels in screenings and corresponding calculations were done as described before [40, 41]. Briefly, fluorescence intensity is related to the cell volume for all data points, resulting in specific eGFP fluorescence. Thereof, the population’s geometric mean is normalized by subtracting background signal (of non-producing *P. pastoris* wild type cells) and related to expression under the control of *P*_*GAP*_. Indel mutation screenings with CRISPR/Cas9-BB3’s were done in a CBS7435(Mut^S^) strain stably expressing eGFP under control of the *P*_*GAP*_ promoter (integration in the native *P*_*GAP*_ locus). Disruption frequency of eGFP (InDel mutations) was analysed by flow cytometry and verified by sequencing of individual clones.

#### InDel mutations using CRISPR/Cas9

Targeting efficiency of the modular CRISPR/Cas9 system on native sequences was evaluated by disruption of the coding sequences of *AOX1* and *DAS1* at two different positions each. CRISPR/Cas9-BB3s plasmids harboring the Cas9/sgRNA transcription units were transformed into electro-competent *P. pastoris* by electroporation (2 kV, 4 ms, GenePulser, BioRad) according to [4] and selected on G418 agar plates. After restreaking the clones two times on selective agar plates the targeted loci were checked for InDel mutations by colony PCR, followed by Sanger sequencing. Sequences of gRNAs and verification primers are listed in Additional file 1: Table S4.

## Additional files

Additional file 1: File contains additional Tables S1-S3. **Table S1.** Gene copy numbers of four GOIs in three engineered *Pichia pastoris* strains after three consecutive batch cultivations. **Table S2.** *P. pastoris* deep-well screening conditions. **Table S3.** Selection markers. **Table S4.** sgRNA sequences for CRISPR/Cas9 and verification primers for InDel mutations. (PDF 277 kb)

Additional file 2: Golden*Pi*CS modules and plasmids. Modules and plasmids are listed with corresponding cloning- and fusion sites and full sequences. DNA orientation is 5’to 3′. All plasmids are available at Addgene. (XLSX 33 kb)

## Abbreviations

BB: Backbone
Cas9: CRISPR-associated protein 9
CRISPR: Clustered Regularly Interspaced Short Palindromic Repeats
eGFP: Enhanced GFP
Fs: Fusion site
GCN: Gene copy number
GGA: Golden Gate Assembly
GOI: Gene of interest
GoldenMOCS: Golden Gate-derived multi-organism cloning system
Golden*Pi*CS: Golden Gate-derived *Pichia pastoris* cloning system
InDel: Insertion or deletion mutation
MUT: Methanol utilization
RNAP: RNA polymerase
sgRNA: Single guide RNA
