# Supplementary material for: GoldenPiCS: a Golden Gate-derived modular cloning system for applied synthetic biology in the yeast Pichia pastoris
Source: BMC Syst Biol. 2017 Dec 8;11:123. doi: 10.1186/s12918-017-0492-3 (PMC5723102; doi:10.1186/s12918-017-0492-3)
Supplement: Supplementary file 1 — File contains additional Tables S1-S3. Table S1. Gene copy numbers of four GOIs in three engineered Pichia pastoris strains after three consecutive batch cultivations. Table S2. P. pastoris deep-well screening conditions. Table S3. Selection markers. Table S4. sgRNA sequences for CRISPR/Cas9 and verification primers for InDel mutations. (PDF 277 kb) [file 12918_2017_492_MOESM1_ESM.pdf]

Supplementary Information:

Table S1: Gene copy numbers of four GOIs in three engineered *Pichia pastoris* strains after three consecutive batch cultivations. Clone 1, 2 and 3 contain 3/3/1/1, 3/3/3/1 and 2/2/2/2 copies of GOI 1-4, respectively. GCN were normalized to the first sample point. Gene copy number analysis was done in triplicates or quadruplicates as described before [40]. Standard deviations were below 7% for all measurements.

	batch 1				batch 2				batch 3			
	GOI1	GOI2	GOI3	GOI4	GOI1	GOI2	GOI3	GOI4	GOI1	GOI2	GOI3	GOI4
clone 1	1	1	1	1	1.0	1.2	1.0	1.0	0.9	1.3	1.0	1.1
clone 2	1	1	1	1	0.9	1.0	0.8	1.0	1.0	1.3	0.9	0.8
clone 3	1	1	1	1	0.9	1.0	0.9	0.9	1.2	1.4	1.1	1.2

Table S2: *P. pastoris* deep-well screening conditions representing specific bioreactor cultivation phases

**Condition**	**ID**	**Start-OD_{600}**	**Cultivation substrate**	**Cultivation time [h]**	**Harvest-OD_{600}**	**Harvest-μ [h^{-1}]**	**Represented bioreactor cultivation phase**
**Excess glycerol**	G	0.1	2% glycerol	15-20 h	3-8	0.23	glycerol batch
**Excess glucose**	D	0.1	2% glucose	15-20 h	3-8	0.23	glucose batch
**Limiting glucose**	X	1	12 mm glucose feed bead	15-20 h	8-12	0.04 (av. 0.14)	glucose fed batch
**Methanol feed**	M	4	0.5% and 1% methanol (at start and after 16h)	16 + 6 h	8-12	0.10	methanol shot/feed

Table S3: Selection markers. Antibiotics, resistance genes and working concentrations for *P. pastoris* and *E. coli* are listed.

**Antibiotic**	**Resistance**	**working concentration *P. pastoris* [μg/mL]**	**working concentration *E. coli* [μg/mL]**
Ampicillin	ampR	100	100
Kanamycin	kanMX	50	50
Geneticin (G418)	kanMX	500	500
Zeocin	zeoR (Sh ble)	25	25
Hygromycin	hphMX	200	50
Nourseothricin	natMX	100	50

Table S4: Sequences of gRNA´s and verification primers for Indel mutations using CRSIPR/Cas9

target	gRNA_1	gRNA_2	Sequencing_primer_fw	Sequencing_primer_rv
eGFP	GGGCGAGGAGCTGTTCACCG	CGCCGGACACGCTGAACTTG	TTGCCCCTTTCACTTGAC	CCTTAACTTGCCAAACCT
AOX1	CTAGGATATCAAACTCTTCG	GACTGTTCCTCAGTTCAAGT	TCTTGGAACCTAATATGACAAAAGC	TTCTTCGCAGGCTTGGCTT
DAS1	AAGCATGACAAGTTGTAAAC	TGGAGAATAATCGAACAAAA	TTCATGTCGCTAAGGCTTTCTT	TTTCAGGTAGCGTCAGATTCGG
